# Polarization of intestinal tumour-associated macrophages regulates the development of schistosomal colorectal cancer

**DOI:** 10.7150/jca.48985

**Published:** 2021-01-01

**Authors:** Zijian Wang, Zhixiang Du, Haoyu Sheng, Xiuliang Xu, Wenjie Wang, Jian Yang, Jian Sun, Jianghua Yang

**Affiliations:** 1 Department of Infectious Diseases, Yijishan Hospital of Wannan Medical College, Wuhu, Anhui 241001, P. R. China.; 2 Department of Infectious Diseases, The People's Hospital of Chizhou, Chizhou, Anhui 247000, P. R. China.

**Keywords:** tumour-associated macrophages, schistosomiasis, colorectal cancer, mechanism, prognosis survival

## Abstract

Tumour-associated macrophages (TAMs) can be divided into M1 and M2 TAMs. M2 TAMs play an important role in tumor progression, promoting a pro-angiogenic and immunosuppressive signal in the tumor. Previous studies have shown a correlation between schistosomiasis and colorectal cancer (CRC), but the specific mechanism has not been clarified. The differences between schistosomal CRC and non-schistosomal CRC were explored by analysing the clinicopathological data and survival time prognosis of schistosomal CRC and non-schistosomal CRC patients. The underlying mechanisms leading to the differences were investigated via tissue pathology experiments. Here, we investigated whether TAMs play a role in schistosomal CRC, leading to different clinicopathological features and prognoses in schistosomal CRC and non-schistosomal CRC patients and whether TAMs have a regulatory effect on the development and prognosis of schistosomal CRC. We found that schistosomal CRC and non-schistosomal CRC patients differ in age, sex, TNM staging and prognosis survival. Applying a logistic regression analysis model, the results showed that age, sex, pathological T stage and combined schistosomiasis were independent risk factors for CRC. Prognostic analysis of follow-up patients with schistosomal CRC found that the T stage, M stage and M2 TAMs numbers were independent prognostic factors for overall survival (OS). TAMs are significantly higher in tissues of schistosomal CRC than in non-schistosomal CRC patients, especially M2 TAMs. Studies on schistosomal colorectal tissue found that the expression of M2 TAMs increased with the malignant process of intestinal tissue. In summary, schistosomal CRC and non-schistosomal CRC patients have different clinicopathological features and prognosis, schistosomiasis is a risk factor for CRC and M2 TAMs are independent prognostic factors for OS.

## Introduction

Schistosomiasis is a disease caused by parasitic flatworms (blood flukes) of the genus *Schistosoma*. Approximately 200 million people worldwide are infected with schistosomes, most of whom are children 1, 2. Adult schistosome worms can escape the human immune system and colonize human blood vessels for prolonged periods. Furthermore, many schistosome eggs can be discharged and deposited in nearby tissues 3. The incidence of schistosomiasis varies by region, and the incidence rate in developing countries is significantly higher compared to developed countries. In countries such as Africa, schistosomiasis is highly prevalent 4. China is also a country with a high prevalence of schistosomiasis. According to reports in 2017, approximately 37,601 people were infected with schistosomal worms in China, which was 30.95% less than the number in 2016. The prevalence of schistosomal disease in China continues to decline, but a rebound in the incidence rates in some areas remains a risk 5.

CRC is a common malignant tumour. In China, the prevalence of CRC has been increasing and has become one of the leading causes of cancer deaths 6. Significantly more male patients have CRC, and differences in CRC also exist between different regions 7. Many risk factors for CRC in Asia, such as age, sex, family history and body fat, have been identified 8. A relationship between schistosomiasis and CRC has been identified by researchers 9, and epidemiological data indicates that a close relationship exists between CRC and schistosomiasis 10. Several studies suggest that long-standing schistosomal colitis is a key factor in the carcinogenic process 8; however, the mechanism underlying the relationship between schistosomiasis and CRC has been poorly studied over the past few decades.

TAMs play an important role in the development of human cancer. TAMs are considered an important part of the tumour microenvironment 11. TAMs can adapt to the stimulatory signals from various organisms by changing their phenotype and function. TAMs can be transformed into M1-type and M2-type TAMs. M1-type TAMs mainly play a role in promoting inflammation, killing bacteria and presenting antigens. In contrast, M2-type TAMs have an inhibitory effect on inflammation and have antiparasitic and tissue-repair effects 12. Here, we investigated the regulation of intestinal TAMs in schistosomiasis CRC tissues.

## Patients and Methods

### Patients' data

Clinical data were obtained from patients who underwent radical resection at the First Affiliated Hospital of Wannan Medical College (Wuhu, P. R. China) from January 2012 to December 2018. Ethics approval was obtained from the Ethics Committee of the First Affiliated Hospital of Wannan Medical College, and informed consent was obtained from all subjects. A retrospective analysis of clinical pathology data of 3554 patients with CRC, including 265 cases of schistosomal and 3289 cases of non-schistosomal CRC, was performed. Logistic regression analysis was used to analyse the risk factors of CRC. At the same time, 43 patients with schistosomal CRC collected in 2012 and 57 non-schistosomal CRC patients obtained by stratified sampling according to their age and degree of differentiation were followed up by telephone to obtain patient data. Univariate and multivariate analyses were performed for the prognostic risk factors for overall survival (OS) in the follow-up patients. All schistosomal CRC patients were chronic schistosomiasis patients from schistosomiasis endemic areas. In the postoperative intestinal histopathological examination, schistosome eggs were found by microscopy. All CRC patients were confirmed by clinical and pathological tests, and patients with other malignant tumours were excluded.

### Experimental samples

Paraffin sections of samples from patients with CRC were collected from the Department of Pathology of the First Affiliated Yijishan Hospital of Wannan Medical College. The patient sections were diagnosed by several experts in the pathology department. The experimental samples were obtained from 3554 CRC patients enrolled at the First Affiliated Yijishan Hospital of Wannan Medical College. The patients were randomly sampled and divided into the CRC group and non-schistosomal CRC group with 50 patients in each group. During the same period, 50 patients with schistosomal enteritis and 50 patients with schistosomal colorectal adenoma were collected. Among the patients with schistosomal CRC, 50 patients with lymph node metastasis and 50 patients without lymph node metastasis were randomly chosen for immunohistochemistry. Ethical approval was obtained from the Ethics Committee of the First Affiliated Yijishan Hospital of Wannan Medical College, and informed consent was obtained from all subjects.

### Immunohistochemical staining

Colorectal samples were fixed in 4% paraformaldehyde. Tissues were embedded in paraffin and cut into 4-μm sections. The sections were submerged in xylene at 40ºC for 30 min for deparaffinization. Deparaffinized tissues were dehydrated in a gradient of alcohol solutions (100%, 95%, 80% and 75%) for 1 min in each. The sections were washed in distilled water for 1 min and then stained with haematoxylin and eosin (HE). After washing three times in PBS for 3 min, the deparaffinized slides were submerged in 120℃ distilled water containing citrate (1: 100) or EDTA (1: 50) for 2 min and incubated for 20 min at room temperature for antigen retrieval. Endogenous peroxidase activity was blocked with 1.0% H_2_O_2_ for 10 min. The sections were incubated with mouse anti-human CD68 (KPI, ab955, Abcam), rabbit anti-human HLA-DR (EPR3692, ab92511, Abcam) and rabbit anti-human CD163 (EPR19518, ab182422, Abcam) monoclonal antibodies at 4°C overnight, followed by incubation with a secondary antibody (Max Vision^TM^ HRP kit-5020, MXB, China). The HRP-conjugated secondary antibody was visualized by development with diaminobenzidine (DAB; DAB-0031/1031, MXB, China). All sections were counterstained with haematoxylin.

### Macrophage counting standard

CD68 antibody-labelled mononuclear TAMs, CD163 antibody-labelled M2 TAMs, and HLA-DR antibody-labelled M1 TAMs were used in this study. M1 and M2 TAMs are two types of macrophages formed by mononuclear TAM polarization. The appearance of yellow-brown particles in the cell membrane or cytoplasm indicated a positive reaction. Under a 400x microscope, positive cells were counted in 5 randomly selected areas with the most positive cells, and the average value was obtained. The same tissue was marked by different antibodies, and the same 5 randomly selected areas were used for each antibody. The Trainable Weka Segmentation plug-in in ImageJ software was used to automatically count positive cells 13, 14, and the data were subjected to statistical analyses.

### Statistical analysis

The data were analysed using SPSS software for Windows (version 20.0; IBM Corp, Armonk, NY, USA). The data are expressed as the frequency (n), percentage (%), mean ± standard deviation (SD) and quartile range. The measurement data were compared using Student's t-test and the Mann-Whitney U test. A chi-square test was used to compare categorical variables. A two-tailed *P* < 0.05 was considered significant. Continuous variables were analysed by Cox regression analysis. Every variable was analysed by univariate analysis to identify all potentially important predictors, and then variables with a *P* ≤ 0.20 in the univariate analysis were included in the multivariable analysis. A multivariable Cox regression analysis was performed to identify predictive factors for OS.

## Results

### Clinicopathological features and logistic multivariate analysis

The clinicopathological data of 3554 patients with CRC were analysed. The average age of the patients with schistosomal CRC was 62.31±11.54 years old, which was significantly lower than that of patients with non-schistosomal CRC (66.34±10.83 years old) (*P* < 0.001). Moreover, significantly more patients over 60 years old were in the schistosomal group compared to the non-schistosomal group (*P*=0.002). Both groups had more males than females, but the proportion of males in the schistosomiasis group was much higher than that in the non-schistosomiasis group (*P*=0.001). In terms of TNM staging, significant differences between the T and M stages of the schistosomiasis group and the non-schistosomiasis group were found (*P*=0.001; *P* < 0.001). The proportion of patients in the schistosomal group in the T1-3 period was 86.79%, which was much higher compared to the non-schistosomal group (77.08%). The proportion of patients in the M1 stage in the schistosomal and non-schistosomal group was 11.32% and 25.42%, respectively. No significant differences were found in the location or N-stage between the two groups (**Table 1**). The variables with *P* < 0.20 in **Table 1** and those deemed to be meaningful in the field were included in the logistic regression analysis model. The results showed that age, sex, pathologic T stage and schistosomiasis status were independent risk factors for CRC (all *P* < 0.05; **Table 2**).

### Univariate and multivariate analysis of the prognostic factors for OS

A univariate analysis was performed on the entire dataset of 43 patients with schistosomal CRC. The results showed that the T stage (*P*=0.011), M stage (*P*=0.001), M2 TAM number (*P*=0.015) and age (*P*=0.068) were significantly correlated with OS. A multivariate analysis was performed to assess the prognostic value of factors with a significant probability of being related to OS (*P* ≤ 0.2 in the univariate analysis; **Table 3**). Factors such as the T stage, M stage and M2 TAM numbers were included in this multivariate analysis. The T stage (*P*=0.029), M stage (*P*=0.013) and M2 TAM numbers (*P*=0.015) were independent prognostic factors for OS.

### TAMs expression in schistosomal and non-schistosomal CRC tissues

**Figure 1:**
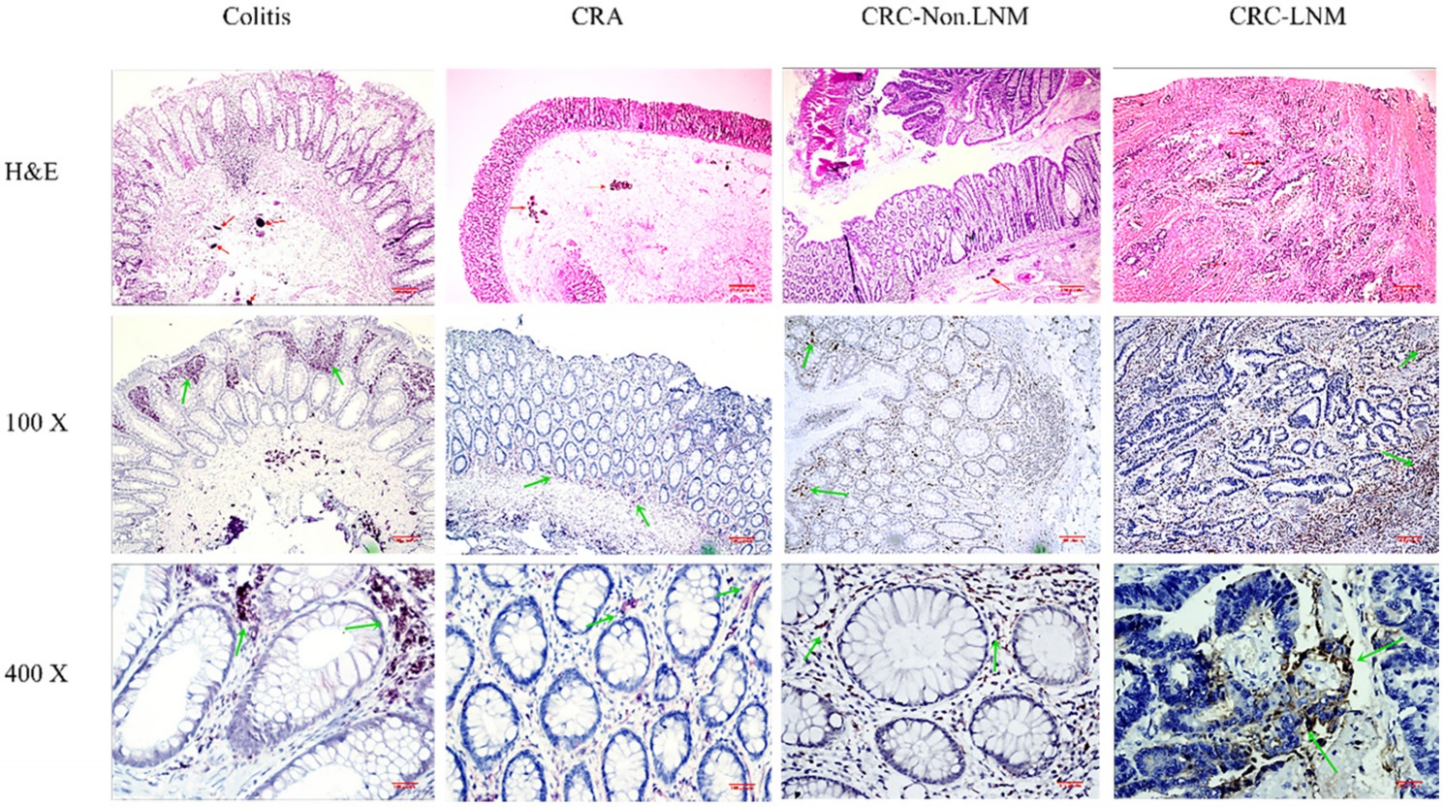
TAMs express differences in CRC SJ and CRC NSJ. A: Expression of CD68-positive TAMs in CRC SJ and CRC NSJ tissues; B: Expression of CD163-positive TAMs (M2 TAMs) in CRC SJ and CRC NSJ tissues. Magnification: x100 and x400. The red arrow marks *Schistosoma japonicum* eggs; the green arrow marks the positive macrophages.

Experimental analyses were performed on CRC tissues with *Schistosoma japonicum* (CRC SJ) and CRC tissues without *Schistosoma japonicum* (CRC NSJ) tissues. The total number of TAMs (CD68 positive) differed significantly between the two groups (schistosomal group, 864.8±31.89; non-schistosomal group, (519.6±42.1; *P* < 0.001; **Table 4**). The number of CD163-labelled M2 TAMs and HLA-DR-labelled M1 TAMs were higher in CRC SJ tissues than in CRC NSJ tissues (all *P* < 0.001; **Fig. 1**).

### Number of macrophages in schistosomal intestinal tissues at different stages

**Figure 2:**
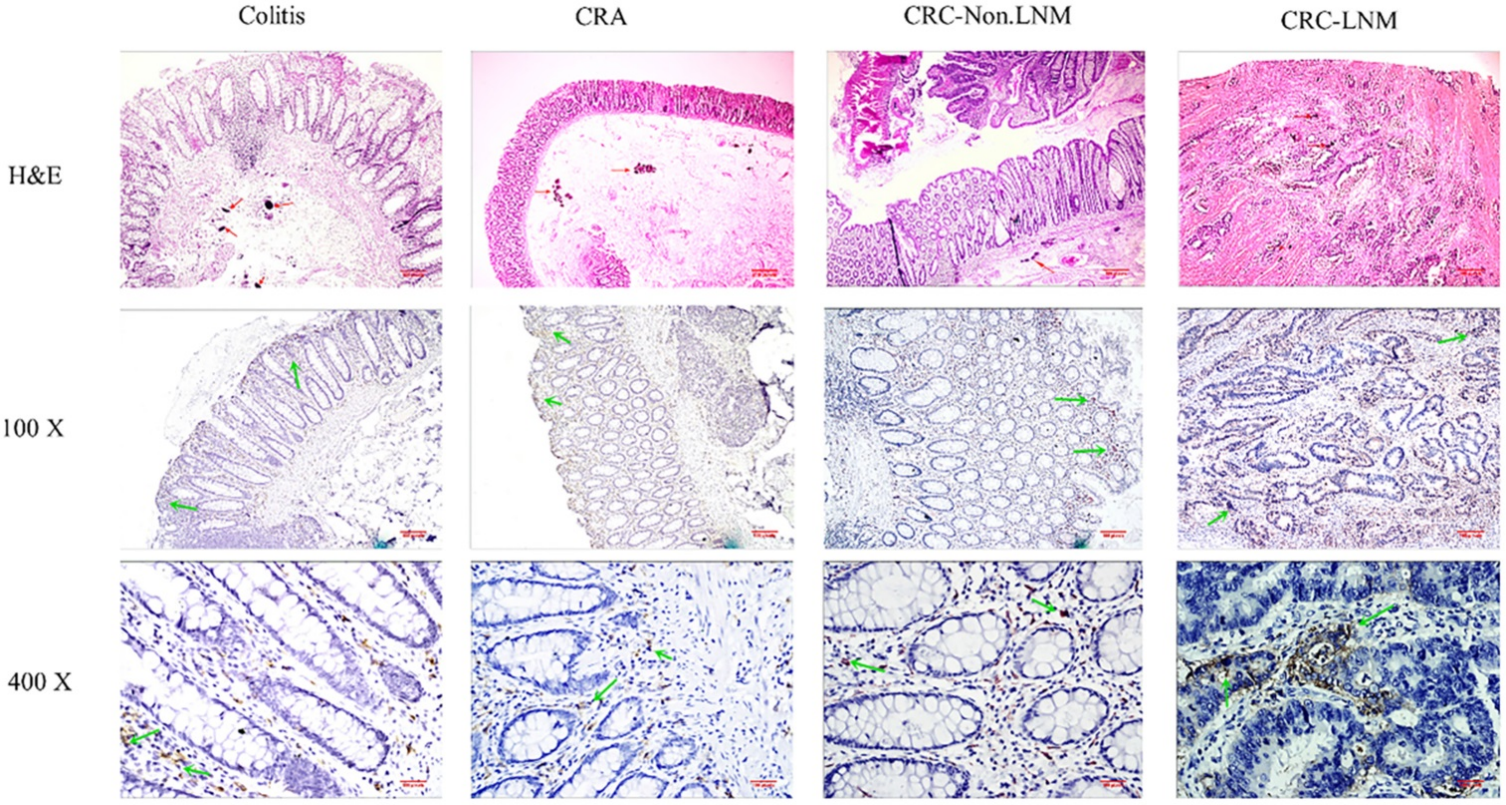
Expression of CD68-positive TAMs (the total number of TAMs) in different stages of intestinal tissues. Magnification: x100 and x400. The red arrow marks *Schistosoma japonicum* eggs; the green arrow marks the positive macrophages.

**Figure 3:**
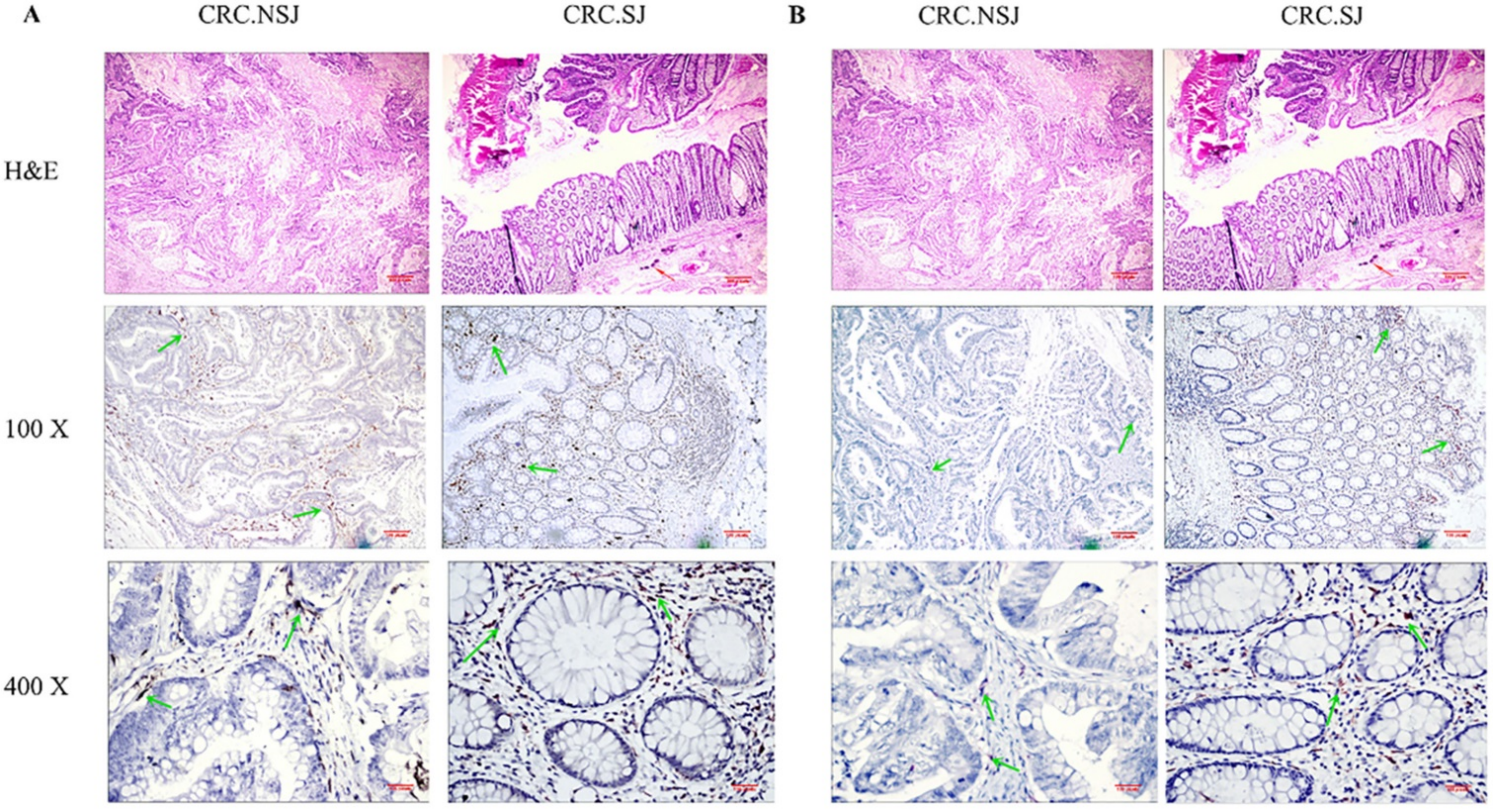
Expression of CD163-positive TAMs (M2 TAMs) in different stages of intestinal tissues. Magnification: x100 and x400. The red arrow marks *Schistosoma japonicum* eggs; the green arrow marks the positive macrophages.

The immunohistochemistry results showed that the total number of macrophages (CD68 positive) was highest at 1221.03±224.6 in inflamed tissues, followed by 1068±34.04 in lymph node metastatic adenocarcinoma (**Table 5**). Overall, the total number of macrophages was increased. The number of CD163-labelled M2 macrophages also tended to increase from adenoma to adenocarcinoma. The number of macrophages in the tissues at different stages significantly differed (all *P* < 0.05). The proportion of CD163/CD68 positive cells obviously increased in different tissues (**Fig. 2 & Fig. 3**).

### TAM expression in lymph node and non-lymph node metastatic cancer tissues

**Figure 4:**
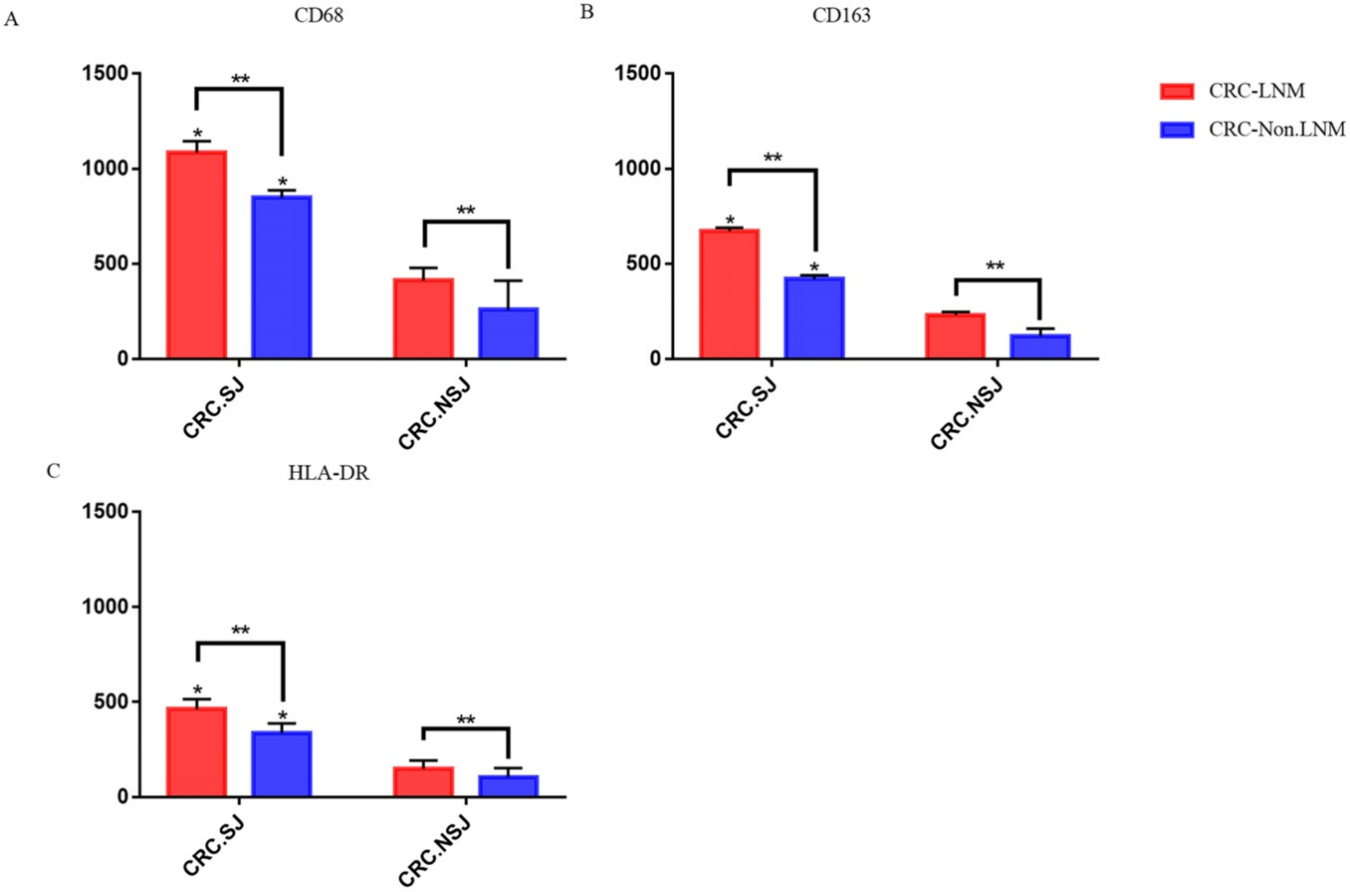
Expression of TAMs in intestinal tissues with or without lymph node metastasis. *: Compared with the non-schistosomiasis group, *P* < 0.05; **: Compared with lymph node distant metastasis, *P* < 0.05.

In patients with schistosomiasis and non-schistosomiasis, the number of M1 and M2 TAMs was higher in patients with lymph node metastases than those without lymph node metastases (*P <* 0.05). The number of TAMs in the schistosomiasis group was significantly higher compared to the non-schistosomiasis group (*P* < 0.05; **Table 6 & Fig. 4**).

## Discussion

Schistosomiasis has a long history spanning more than 2100 years, as evidenced by the discovery of eggs in the liver of an ancient corpse. Schistosomiasis in China is mainly caused by *S. japonicum* infections 15. Schistosomiasis is a chronic parasitic disease that is often neglected because it mainly affects poor rural communities in developing counties 16. In recent years, several studies have suggested that the occurrence of CRC is associated with infections with bacteria 17, 18, parasites 19, 20 and other pathogens.

In this study, we analysed the characteristics of CRC patients with and without schistosomiasis. A total of 3554 CRC patients were enrolled, including 3289 CRC patients without chronic schistosomiasis and 265 CRC patients with chronic schistosomiasis. Our clinicopathological evidence and survival time analysis showed significant differences in age, sex, pathologic T stage, pathologic M stage and prognosis survival time between patients with schistosomal CRC and non-schistosomal CRC. The average age of schistosomal CRC patients was significantly higher compared to non-schistosomal CRC patients (*P* < 0.001). Moreover, the proportion of patients older than 60 years in the schistosomal group was significantly higher compared to the non-schistosomal group (*P*=0.002). Both groups had more males than females, but the proportion of males in the schistosomal group was significantly higher compared to the non-schistosomal group (*P*=0.001), which may be due to differences in water exposure or diet 21, 22. Studies have shown that dietary intakes differ between women and men 23. The ratio of males to females in the schistosomal group was 2.3, which was higher than that of the non-schistosomal group (1.4), which may be related to infection with schistosomiasis. Past studies have shown that men are twice as likely to have schistosomiasis as women 24, and is similar to the male-female ratio of schistosomal CRC patients in this paper. Schistosomiasis may affect the proportion of male to female patients with CRC 25, 26. In terms of TNM staging, the proportion of T1-3 patients in the schistosomal group was 75.85%, and the proportion of T4 patients was 24.15%. The proportion of T1-3 patients in the non-schistosomal group was 77.08%, and the proportion of T4 patients was 22.92%. The proportion of T4 patients in the schistosomal group was significantly higher compared to the non-schistosomal group (*P*=0.02). The proportion of M1 phase patients in the schistosomal group was 25.28% (the proportion of M0 phase patients was 74.72%), which was significantly higher than the non-schistosomal group (11.31%; the proportion of M0 phase patients was 88.69%). These data show that the degree of malignancy of schistosomiasis patients is higher compared to non-schistosomiasis patients, which may be due to mechanistic changes in the development of CRC caused by long-term schistosomiasis infection 27. The logistic regression analysis showed that age, sex, pathologic T stage and schistosomiasis were independent risk factors for colorectal cancer (all *P* < 0.05). This result is consistent with the report by Regula 28. Schistosomiasis and CRC have a certain correlation 29, 30. The analysis of prognostic factors in the schistosomiasis group indicated that the number of M2 TAMs is an independent prognostic risk factor for schistosomal CRC, which explains the difference in prognosis survival time between patients with schistosomal CRC and those with non-schistosomal CRC.

The immunohistochemical staining results indicated that the mean numbers of TAMs in patients with colitis were significantly higher than those in CRC patients, and the mean numbers of TAMs in patients with schistosomiasis were significantly higher than those in patients without schistosomiasis. The total number of TAMs significantly decreased from inflamed tissues to tumour tissues, which may be related to the pro-inflammatory function of M1 TAMs 31-36. In summary, our data indicate that the decrease in TAMs is mainly caused by a decrease in M1 macrophages. With the progression of CRC, the number of TAMs rises significantly, especially M2 TAMs. TAMs polarization plays a pivotal role in the development of CRC in which M2 TAMs play a leading role. Schistosomiasis may alter the mechanism underlying the development and progression of CRC, leading to changes in its clinicopathological features and prognosis 37. TAMs may play an important role in this process 38-42. In this study, we found that the number of TAMs and the proportion of M2 TAMs in schistosomal CRC tissues were much higher than that in non-schistosomal CRC tissues; both differences were significant. Cortes-Selva D found that schistosomiasis can induce a strong Th2-biased immune response in the host and polarize macrophages into a broad M2 phenotype, consistent with the results reported here 43. The number of TAMs continues to rise during tumour development, and the proportion of M2 macrophages increases significantly. As tumours develop, schistosomiasis may promote the polarization of TAMs to M2 TAMs. Gong W found that excretory-secretory (ES) antigens derived from *S. japonicum* eggs can activate macrophages, which exhibit M2 polarization 44. TAMs polarization may be a potential mechanism for the development of schistosomal CRC. We also found that the number of TAMs in patients with lymph node metastases was significantly higher than that in patients without distant lymph node metastases. The schistosomal group was markedly different from the non-schistosomal group. Previous studies have found that a high density of TAMs in the margin of tumour invasion has a positive effect on the prognosis of patients with CRC, and a high density of M2 TAM infiltration in the tumour centre is a powerful indicator of poor prognosis 45. TAMs are related to the development and prognosis of CRC 46, 47.

## Conclusions

Schistosomiasis may affect the occurrence and development of CRC 48, 49. We report here that in patients with schistosomal CRC, schistosomiasis may promote the development of CRC by affecting the transformation of TAMs to the M2 phenotype. The mechanism of cancer development in patients with schistosomiasis versus non-schistosomiasis may be altered, thereby changing the clinicopathological features and prognosis of these two groups of patients.

## Figures and Tables

**Table 1:** Clinical pathological characteristics of patients'

Characteristics	n (%), or mean ± SD		*P*-value
	CRC SJ	CRC NSJ	
	n = 265	n = 3289	
**Age, years**	62.31±11.54	66.34±10.83	< 0.001
< 60	77 (29.06)	1276 (38.80)	0.002
≥ 60	188 (70.94)	2013 (61.20)	
**Sex**			0.001
Male	185	1946	
Female	80	1343	
**Location**			0.165
Right colon	50 (18.87)	742 (22.56)	
Left colon	215 (81.13)	2547 (77.44)	
**Differentiation**			0.479
Poor	7 (2.64)	80 (2.43)	
Moderate	249 (93.96)	3135 (95.32)	
Well	9 (3.40)	74 (2.25)	
**Pathologic T stage**			0.02
T1-2	35 (13.21)	667 (20.28)	
T3	166 (62.64)	1868 (56.80)	
T4	64 (24.15)	754 (22.92)	
**Pathologic N stage**			0.091
N0	188 (70.94)	2400 (72.97)	
N1	56 (21.13)	552 (16.78)	
N2	21 (7.93)	337 (10.25)	
**Pathologic M stage**			< 0.001
M0	198 (74.72)	2917 (88.69)	
M1	67 (25.28)	372 (11.31)	

CRC SJ: Colorectal cancer with *Schistosoma japonicum*; CRC NSJ: Colorectal cancer without *Schistosoma japonicum.*

**Table 2:** Multivariate logistic regression analysis of risk factors for colorectal cancer

Variable	β	SE	Wald	*P*	*OR*	95%CI
Age (Years)	0.295	0.081	3.642	0.003	1.335	(1.143, 1.572)
Gender	0.632	1.213	0.521	0.002	1.644	(1.163, 2.356)
Tumor Location	-34.075	0.744	-45.80	0.994	0.0001	(0.681, 2.877)
Pathologic T stage	0.877	0.197	4.452	0.005	2.404	(1.296, 4.458)
Pathologic M stage	1.379	0.912	1.512	0.170	3.968	(0.560, 2.103)
Schistosomiasis	0.546	0.574	0.904	0.029	1.726	(0.579, 2.048)

**Table 3:** Univariate and multivariate analysis of the prognostic factors for overall survival (OS)

Characteristics	Univariate analysis	Multivariate analysis		
	*P*-Value	*P*-Value	HR	95% CI
Age, years	0.068	0.543	-	-
Gender	0.574	-	-	-
Location	0.823	-	-	-
Differentiation	0.155	0.761	-	-
Histological type	0.231	-	-	-
**Pathologic T stage**	0.011	0.029		
T1-2			0.477	0.092-2.473
T3			1.191	0.139-10.206
T4			3.555	1.155-10.941
**Pathologic N stage**	0.091	0.132	-	-
N0			-	-
N1			-	-
N2				
**Pathologic M stage**	0.001	0.013		
M0			0.327	0.0125-1.873
M1			2.562	1.673-3.795
**Macrophages**				
M1	0.216	-	-	-
M2	0.015	0.025	2.132	0.145-11.358

**Table 4:** TAMs expression in CRC.SJ and CRC.NSJ tissues

	CRC SJ	CRC NSJ	*P*-value
CD68	837.6±12.4	528.9±13.18	< 0.001
CD163	423.3±8.933	231.7±5.659	< 0.001
HLA-DR	331.8±11.52	189.5±5.992	< 0.001
CD163/CD68	0.51	0.44	

CRC SJ: Colorectal cancer with *schistosoma japonicum*; CRC NSJ: Colorectal cancer without *schistosoma japonicum.*

**Table 5:** Expression of TAMs in different stages of intestinal tissue of schistosomiasis

	Colitis	CRA	CRC-Non.LNM	CRC-LNM
CD68	1221.03±224.6	631.2±63.42	864.8±31.89	1068±34.04
CD163	489.3±148.2	290±38.41	418.8±12.07	471.3±10.44
HLA-DR	732.5±124.3	245.7±10.03	338.3±28.75	462.7±30.61
CD163/CD68	0.4	0.46	0.48	0.63

CRA: Colorectal adenoma; CRC-NonLNM: Colorectal cancer without lymph node distant metastasis; CRC-LNM: Colorectal cancer with distant lymph node metastasis; Comparison between groups *P* < 0.05.

**Table 6:** Expression of TAMs in cancer tissues of patients with colorectal cancer with or without lymph node metastasis

	CRC-NonLNM		CRC-LNM	
	With SJ	Without SJ	With SJ	Without SJ
CD68	864.8±31.89^ab^	257.6±88.46^b^	1068±34.04^a^	413.58±37.42
CD163	418.8±12.07^ab^	121.7±20.95^b^	671.3±10.44^a^	230.53±9.72
HLA-DR	338.3±28.75^ab^	105±27.83^b^	462.7±30.61^a^	149.47±25.05

With SJ: with Schistosoma japonicum; Without SJ: without Schistosoma japonicum. a: Compared with the non-schistosomiasis group *P* < 0.05; b: Compared with lymph node distant metastasis *P* < 0.05.

## Abbreviations

TAMs: Tumour-associated macrophages
CRC: Colorectal cancer
CRA: Colorectal adenoma
CRC-LNM: Colorectal cancer with distant lymph node metastasis
CRC-NonLNM: Colorectal cancer without lymph node distant metastasis
OS: Overall survival
CRC SJ: CRC tissues with *Schistosoma japonicum*
CRC NSJ: CRC tissues without *Schistosoma japonicum*
